# Iron-responsive transcription factor SreA regulates calcium homeostasis independently of calcineurin–Crz1 pathway in *Fusarium graminearum*

**DOI:** 10.1128/spectrum.01537-26

**Published:** 2026-08-18

**Authors:** Heeji Moon, Jiyeun Park, Soobin Shin, Sieun Kim, Jung-Eun Kim, Hokyoung Son

**Affiliations:** 1 Department of Agricultural Biotechnology, Seoul National University 26725, Seoul, Republic of Korea; 2 Research Institute of Agriculture and Life Sciences, Seoul National University 26725, Seoul, Republic of Korea; 3 The Sainsbury Laboratory, Norwich, United Kingdom; 4 Agricultural Microbiology Division, National Institute of Agricultural Sciences, Rural Development Administration 230986, Wanju, Republic of Korea; 5 Horticultural and Herbal Crop Environment Division, National Institute of Horticultural and Herbal Science, Rural Development Administration 588019, Wanju, Republic of Korea; 6 Plant Health Center, Seoul National University 26725, Seoul, Republic of Korea; 7 Plant Genomics and Breeding Institute, Seoul National University 26725, Seoul, Republic of Korea; Agroscope, Nyon, Switzerland

**Keywords:** *Fusarium graminearum*, calcium homeostasis, iron homeostasis, transcription factor

## Abstract

Calcium is an abundant intracellular ion that is intricately involved in various cellular processes. The precise regulation of calcium homeostasis is critical for fungal physiology. In fungi, calcium signaling is primarily mediated by the calmodulin–calcineurin pathway, with the transcription factor Crz1 acting as a key downstream regulator of calcium homeostasis. However, other transcription factors that contribute to calcium homeostasis in *Fusarium graminearum* are yet to be systematically identified. Here, we screened 675 transcription factor deletion mutants of *F. graminearum* under calcium stress and identified eight mutants, including *crz1* and *sreA*. We found that SreA, a known iron homeostasis regulator, is required for calcium stress tolerance, separate from its role in iron homeostasis. Furthermore, SreA functions independently of the calcineurin–Crz1 pathway, and deletion of *SREA* alters cytosolic Ca^2+^ dynamics following calcium stimulation. Our findings highlight a previously unrecognized role of SreA in fungal calcium homeostasis and suggest that SreA contributes to calcium stress response by maintaining appropriate cytosolic Ca^2+^ signaling in *F. graminearum*.

**IMPORTANCE:**

Calcium regulation is vital for the survival and pathogenicity of fungi. While the transcription factor Crz1 is widely recognized as the main regulator of calcium homeostasis, this study reveals that another transcription factor, SreA, plays a critical and previously unrecognized role in *Fusarium graminearum*. Although SreA is a well-established regulator of iron homeostasis, it also plays a critical role in calcium stress tolerance. This function is independent of both iron homeostasis and the canonical calcineurin–Crz1 pathway. Together, these findings broaden our understanding of the transcriptional regulators involved in fungal calcium homeostasis.

## INTRODUCTION

Calcium ions (Ca^2+^) function as ubiquitous secondary messengers regulating a wide range of cellular processes. Transient changes in cytosolic Ca^2+^ levels are rapidly induced in response to environmental and physiological stimuli, playing central roles in coordinating cellular physiology, stress adaptation, and development (1, 2). Upon stimulation, elevated cytosolic Ca^2+^ is sensed by the calcium-binding protein calmodulin, which activates the conserved serine/threonine phosphatase calcineurin to coordinate downstream signaling involved in calcium homeostasis, stress adaptation, and development (3, 4).

The zinc finger transcription factor Crz1, acting downstream of calcineurin, is a well-characterized regulator of calcium homeostasis in fungi (5). Upon dephosphorylation by calcineurin, Crz1 translocates into the nucleus, where it regulates the expression of genes involved in calcium transport, ion homeostasis, and stress adaptation (5–7). In *Saccharomyces cerevisiae*, Crz1 controls calcium homeostasis by regulating genes encoding plasma membrane Ca^2+^ channels and vacuolar Ca^2+^ pumps, while also coordinating broader transcriptional programs associated with cell wall integrity and ion balance (8, 9). In the rice blast fungus *Magnaporthe oryzae*, the ortholog MoCrz1 maintains intracellular Ca^2+^ levels by inducing the expression of P-type Ca^2+^-ATPases, such as *PMC* and *PMR*, while also coordinating downstream pathways required for host penetration and virulence (10, 11). Similarly, in *Aspergillus fumigatus* and *Aspergillus nidulans*, the homolog CrzA coordinates transcriptional programs required for calcium tolerance, cell wall organization, and adaptation to cation and cell wall stresses (7, 12, 13). Furthermore, Crz1 homologs in other fungal pathogens, such as *Candida albicans*, *Penicillium digitatum*, and *Botrytis cinerea*, have been implicated in ion stress tolerance and the maintenance of cell wall and membrane integrity, while also contributing to antifungal drug tolerance (14–17). Taken together, these studies establish Crz1 as a major regulator of calcium homeostasis and adaptive stress responses in fungi.

Although Crz1 plays a well-established role in fungal calcium homeostasis, multiple regulatory mechanisms operate alongside it to coordinate intracellular Ca^2+^ responses. In *Cryptococcus neoformans*, calcineurin regulates stress adaptation not only through Crz1-dependent transcription but also via Puf4 and Pbp1, which mediate post-transcriptional control in parallel pathways (18, 19). In addition, several transcription factors that contribute to calcium homeostasis have been identified in *A. fumigatus*. For instance, the transcription factor ZipD responds to calcium in a calcineurin-dependent manner (20). ZipD regulates calcium metabolism by sharing downstream genes with CrzA, while also contributing to cell wall organization, osmotic stress tolerance, and fungicide resistance. Similarly, ZnfA acts collaboratively with CrzA in modulating the response to calcium, cell wall, and osmotic stresses (21). Furthermore, SltA functions as a key transcriptional regulator of calcium homeostasis under calcium-limiting conditions in *A. fumigatus*, where it regulates genes involved in calcium metabolism (22). In *A. nidulans*, SltA likewise exerts regulatory effects opposite to those of CrzA on the expression of a vacuolar Ca^2+^/H^+^ exchanger *VCX1* (12). Collectively, these findings highlight the involvement of multiple regulatory factors in fungal calcium homeostasis.

*Fusarium graminearum*, the causal agent of Fusarium head blight (FHB), is a destructive plant pathogen that affects wheat, barley, and other cereals worldwide (23–25). FHB epidemics result in severe yield losses and contaminate grain with mycotoxins, such as deoxynivalenol, posing significant threats to food safety and public health (26). Disease management is further complicated by the limited availability of resistant cultivars and the emergence of resistance to fungicides, highlighting the importance of elucidating the cellular regulatory mechanisms that underlie fungal adaptation and pathogenicity in this pathogen (27–29).

To date, several studies have investigated calcium signaling in *F. graminearum* (30, 31). Fg03141, hereafter referred to as Crz1, has been identified as a Crz1 ortholog that exhibits calcineurin-dependent nuclear localization and, similar to its orthologs in other fungi, plays a critical role in tolerance to antifungal agents (31). Nevertheless, the extent to which additional transcriptional regulators participate in calcium stress responses beyond this canonical pathway is still poorly understood in this fungus. To address this gap, we systematically screened 675 transcription factor deletion mutants of *F. graminearum* under calcium stress conditions and identified a subset of mutants, including *crz1* and *sreA*, that displayed increased calcium sensitivity. Based on these findings, we focused on SreA, a GATA-type transcription factor known as a key regulator of iron homeostasis, and aimed to investigate its role in fungal calcium homeostasis and its relationship with the calcineurin–Crz1 pathway in *F. graminearum*.

## RESULTS

### SreA, an iron-responsive transcription factor, contributes to Ca^2+^ stress tolerance in *F. graminearum*

**Fig 1:**
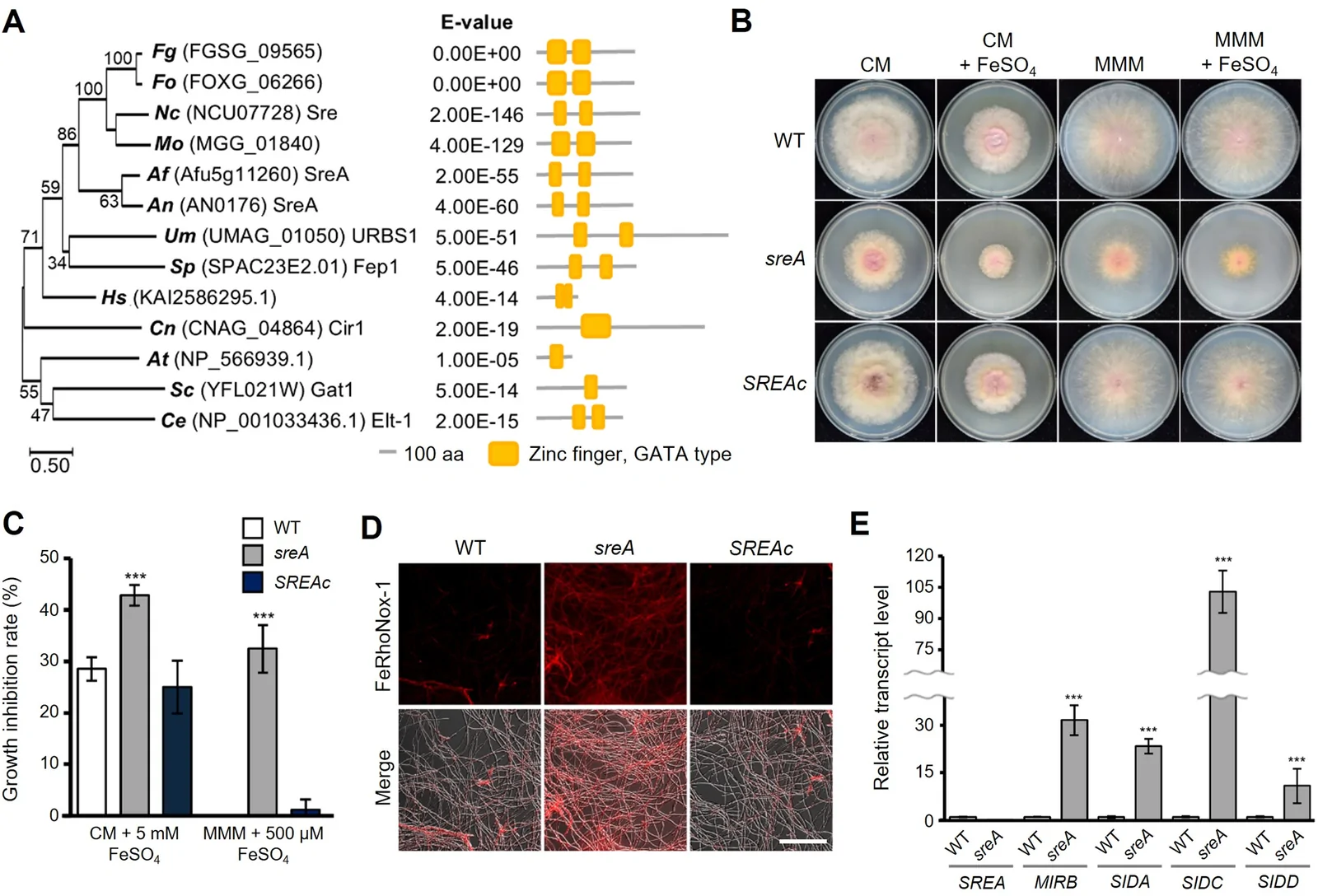
SreA regulates iron homeostasis in *Fusarium graminearum*. (**A**) Phylogenetic tree of SreA orthologs. Amino acid sequences were aligned using ClustalW, and phylogenetic analysis was performed using the maximum likelihood method in MEGA 11. *Fg*, *Fusarium graminearum*; *Fo*, *Fusarium oxysporum*; *Nc*, *Neurospora crassa*; *Mo*, *Magnaporthe oryzae*; *Af*, *Aspergillus fumigatus*; *An*, *Aspergillus nidulans*; *Um*, *Ustilago maydis*; *Sp*, *Schizosaccharomyces pombe*; *Cn*, *Cryptococcus neoformans*; *Ce*, *Caenorhabditis elegans*; *Hs*, *Homo sapiens*; *Sc*, *Saccharomyces cerevisiae*; *At*, *Arabidopsis thaliana.* (**B**) Sensitivity of the wild-type, *sreA*, and complemented strain *SREA*c to FeSO_4_. Mycelial plugs of each strain were inoculated onto complete medium (CM) or MMM, either without FeSO₄ or supplemented with 5 mM or 500 μM FeSO₄ and incubated at 25°C for 5 days. (**C**) Quantification of mycelial growth inhibition under FeSO₄ treatment. Error bars represent standard deviation (***P < 0.001; Student’s *t*-test). (**D**) Measurement of intracellular iron levels. Iron accumulation in wild type, *sreA*, and *SREA*c was detected using 5 μΜ FeRhoNox-1, a fluorescent iron-binding dye, after cultivation in CM at 25°C for 36 h. Images were acquired using a laser scanning microscope. Scale bar = 10 µm. (**E**) Expression of iron acquisition genes. Relative transcription levels of genes involved in iron homeostasis in wild type and *sreA* cultured in MMM for 24 h. Asterisks indicate significant differences compared with wild type (***P < 0.001; Student’s *t*-test).

To identify transcription factors (TFs) involved in calcium homeostasis in *F. graminearum*, we screened a previously constructed library of 675 TF deletion mutants under mild calcium stress conditions (10 mM CaCl_2_) (32). This condition was established based on the calcium-sensitive phenotype of the previously characterized *crz1* mutant, with the rationale that transcription factors important for calcium homeostasis would exhibit detectable growth defects even under mild calcium stress. Under this condition, eight TF mutants, including the *crz1* mutant, exhibited altered sensitivity compared to the wild type on modified minimal medium (MMM) supplemented with CaCl_2_ (Fig. S1). Among them, we focused on the ΔFGSG_09565 mutant, which displayed the most pronounced sensitivity to calcium stress. Phylogenetic analysis revealed that FGSG_09565 is an ortholog of SreA, an iron-responsive GATA transcription factor of *Neurospora crassa*, *Aspergillus fumigatus*, and *A. nidulans* (Fig. 1A). To confirm that the observed calcium-sensitive phenotype was caused by deletion of *SREA*, we generated a complementation strain (*SREA*c). Under enhanced calcium stress (100 mM CaCl_2_), the mutant exhibited pronounced growth inhibition compared with the wild-type strain, and this phenotype was fully restored in the complementation strain (Fig. S1).

SreA was previously identified as a regulator of iron homeostasis in *F. graminearum* (33). To confirm that the *sreA* mutant generated in this study recapitulated the previously reported defects in iron homeostasis, we assessed its growth under iron stress conditions, intracellular iron accumulation, and expression of iron acquisition genes. Compared to the wild-type strain, the *sreA* mutant exhibited increased sensitivity under iron-replete conditions on both complete medium (CM) and MMM supplemented with FeSO_4_, and these phenotypes were fully restored in the complementation strain (Fig. 1B and C). Intracellular iron levels were measured using the fluorescent iron-binding dye FeRhoNox-1. The *sreA* mutant accumulated significantly higher levels of intracellular iron compared to the wild-type strain (Fig. 1D; Fig. S2A). Quantitative reverse transcription-PCR (qRT-PCR) analysis consistently showed that the expression of iron acquisition genes, including the siderophore transporter *MIRB* and siderophore biosynthesis genes *SIDA*, *SIDC*, and *SIDD*, was markedly upregulated in the *sreA* mutant (Fig. 1E). Together, these results confirm that the *sreA* mutant generated in this study faithfully recapitulates the previously reported iron-related phenotypes of SreA in *F. graminearum*.

### Calcium stress sensitivity of the *sreA* mutant is independent of intracellular iron accumulation

**Fig 2:**
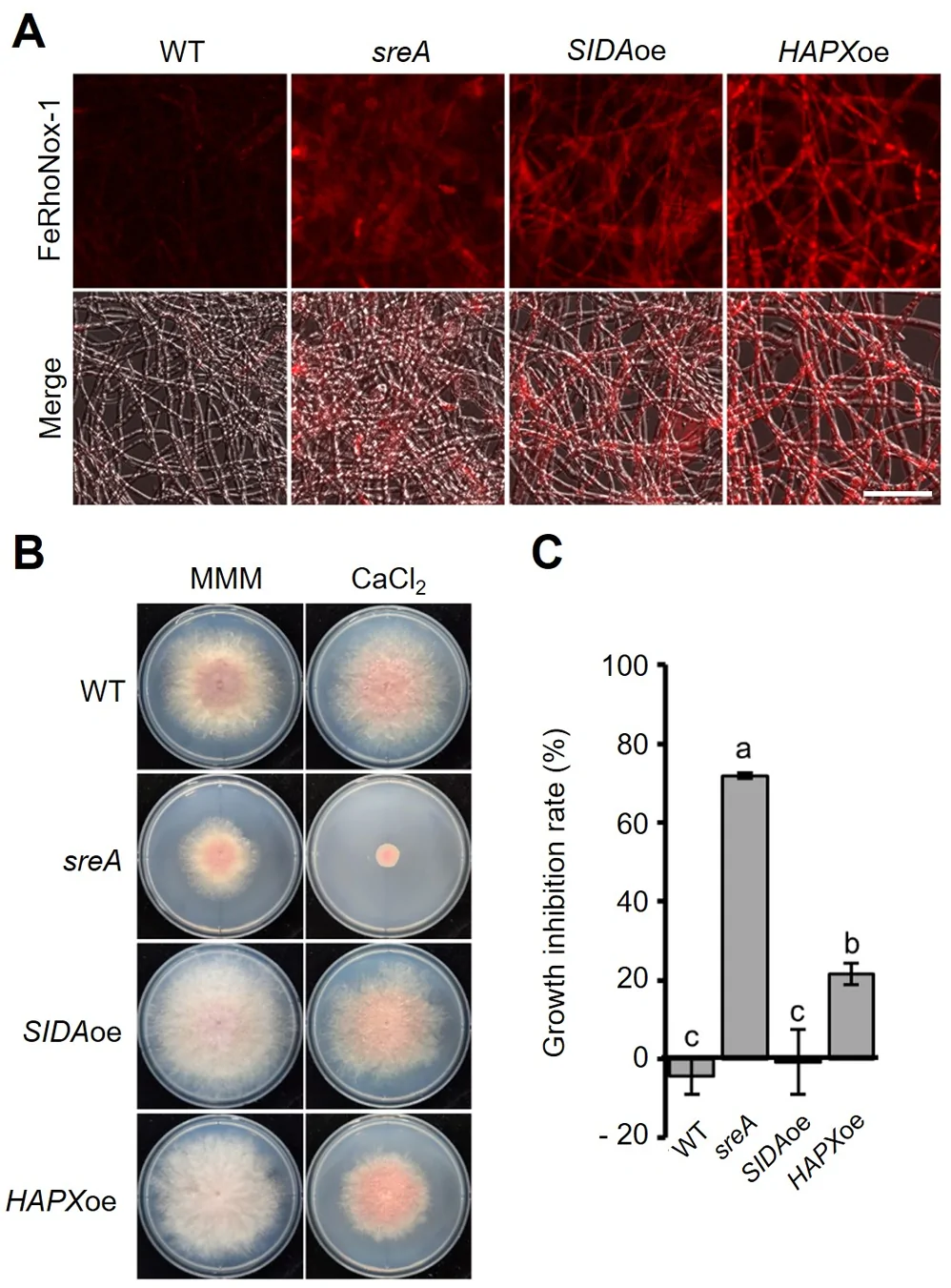
Iron accumulation and calcium stress sensitivity of the wild-type, *sreA*, *SIDA*oe, and *HAPX*oe strains. (**A**) Measurement of intracellular iron levels. Iron accumulation in wild type, *sreA, SIDAoe*, and *HAPXoe* was detected using 5 μM FeRhoNox-1, after cultivation in CM at 25°C for 36 h. Images were acquired using a laser scanning microscope. Scale bar = 10 µm. (**B**) Sensitivity of wild-type, *sreA, SIDAoe*, and *HAPXoe* strains to calcium stress. Mycelial plugs of each strain were inoculated onto MMM with or without 100 mM CaCl_2_ and incubated at 25°C for 5 days. (**C**) Statistical analysis of mycelial growth inhibition under calcium stress. Different letters indicate significant differences between strains based on one-way analysis of variance (ANOVA), followed by Bonferroni post hoc test (*P* < 0.01).

To determine whether the elevated iron accumulation observed in the *sreA* mutant is the direct cause of its calcium sensitivity, we generated *SIDA* and *HAPX* overexpression mutants*,* designated as *SIDA*oe and *HAPX*oe, respectively. *SIDA* encodes a siderophore biosynthesis enzyme repressed by SreA under iron-replete conditions, whereas HapX is known to repress *SREA* expression under iron-limited conditions. Fluorescence analysis using FeRhoNox-1 revealed elevated intracellular iron levels in both overexpression mutants compared to the wild-type strain (Fig. 2A; Fig. S2B).

We next examined the growth of these mutants under calcium stress conditions. The *sreA* mutant exhibited approximately 70% growth inhibition under calcium stress (Fig. 2B and C). In contrast, the *SIDA*oe mutant did not show growth inhibition, displaying growth comparable to that of the wild-type strain under the tested levels of calcium stress. Although the *HAPX*oe mutant exhibited increased sensitivity to calcium stress, the growth inhibition was limited to approximately 20%, substantially lower than that observed in the *sreA* mutant. These results indicate that elevated intracellular iron accumulation does not account for the calcium-sensitive phenotype associated with loss of *SREA*.

### SreA functions independently of the calcineurin–Crz1 signaling pathway

**Fig 3:**
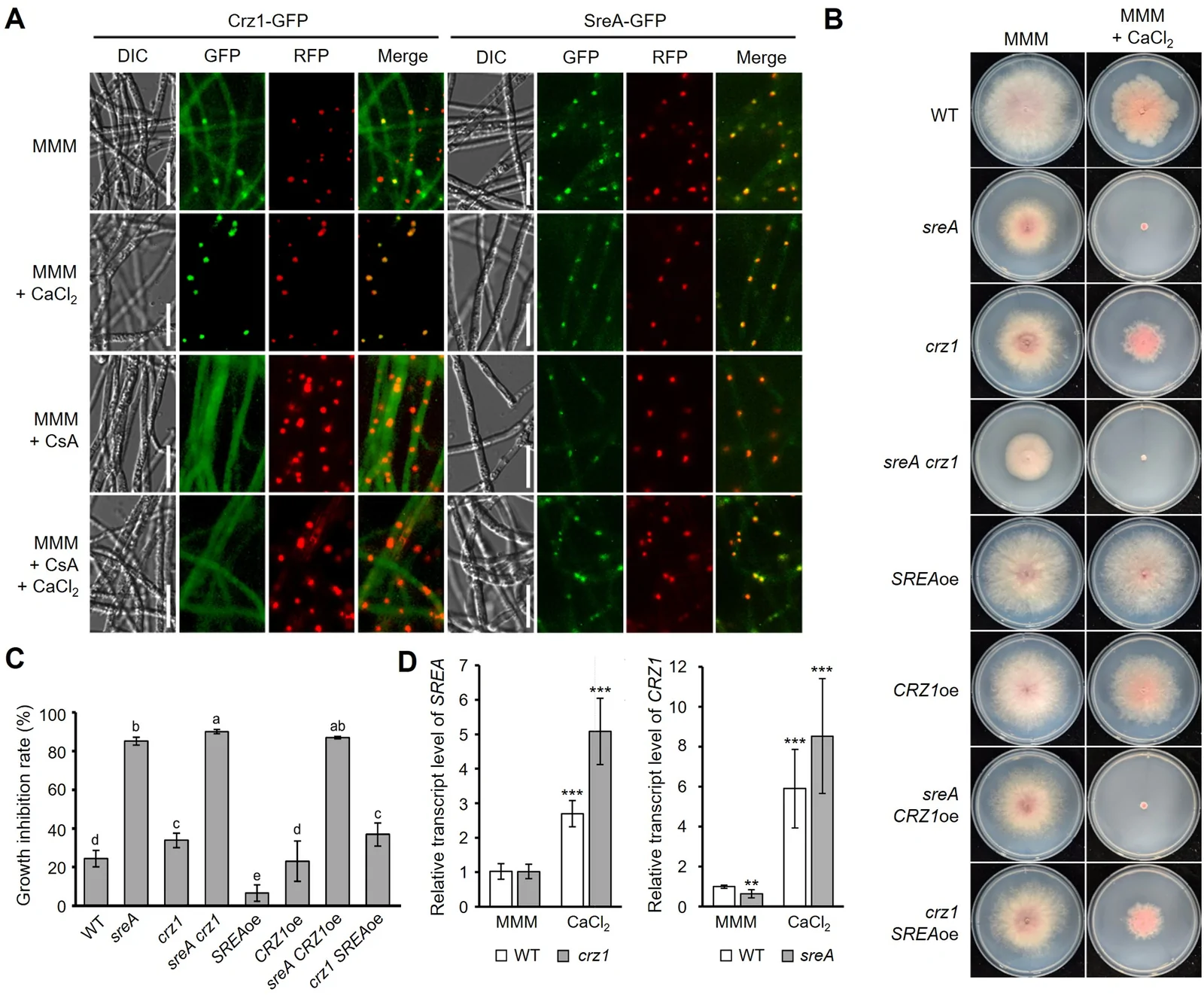
Genetic interaction between SreA and Crz1. (**A**) Subcellular localization of SreA-GFP and Crz1-GFP. Cells were treated with CaCl_2_ and/or 50 μM CsA for 30 min. Scale bar = 25 µm. (**B**) Mycelial growth of the wild-type, *sreA*, *crz1*, and corresponding overexpression strains under calcium stress conditions. Mycelial plugs were inoculated onto MMM with or without 100 mM CaCl_2_ and incubated at 25°C for 5 days. (**C**) Statistical analysis of mycelial growth inhibition under calcium stress. Different letters indicate significant differences based on one-way ANOVA, followed by Bonferroni post hoc test (*P* < 0.01). (**D**) Relative transcript level of *SREA* and *CRZ1* in the *crz1* and *sreA* mutant, respectively. Statistical significance was determined by Student’s *t*-test and is indicated by asterisks (**P < 0.01; ***P < 0.001).

Calcineurin is a Ca^2+^/calmodulin-dependent phosphatase that serves as a central regulator of calcium signaling in fungi. Several transcriptional regulators involved in fungal calcium homeostasis, including Crz1, function in a calcineurin-dependent manner. To determine whether SreA is involved in calcium homeostasis through this canonical pathway, we generated strains expressing GFP-tagged Crz1 and SreA, and then examined their subcellular localization in response to calcium stress and the calcineurin-specific inhibitor cyclosporin A (CsA). Consistent with previous reports (3, 31), Crz1 translocated to the nucleus upon calcium treatment, and this translocation was abolished by CsA treatment, confirming that Crz1 is regulated by calcineurin (Fig. 3A). In contrast, SreA localized to the nucleus under both control and calcium-treated conditions, and its nuclear localization was unaffected by CsA treatment. These results indicate that SreA functions independently of the calcineurin–Crz1 signaling pathway.

Although SreA operates independently of direct calcineurin regulation, we next investigated the genetic relationship between SreA and Crz1 in the calcium stress response. To examine whether these two regulators function in either a common or parallel pathway, we generated a *sreA crz1* double deletion mutant, as well as strains in which *CRZ1* was overexpressed in the *sreA* mutant and *SREA* was overexpressed in the *crz1* mutant. As previously observed, each single deletion mutant exhibited increased sensitivity to calcium stress compared to the wild-type strain, with the *sreA* mutant showing approximately 80% growth inhibition and the *crz1* mutant showing approximately 30% growth inhibition. Notably, the double deletion mutant displayed significantly enhanced sensitivity compared to either single deletion mutant (Fig. 3B and C). Furthermore, while overexpression of *SREA* (*SREA*oe) in the wild-type strain reduced calcium sensitivity, resulting in only ~7% growth inhibition under calcium stress, it failed to rescue the sensitive phenotype of the *crz1* mutant. Similarly, overexpression of *CRZ1* had no effect on the calcium sensitivity of the *sreA* mutant. These results demonstrate that SreA and Crz1 operate through distinct, non-redundant pathways that additively contribute to the calcium stress response in *F. graminearum*.

To further examine potential compensatory transcriptional responses, we analyzed the transcript levels of *SREA* in the *crz1* deletion mutant and *CRZ1* in the *sreA* deletion mutant under calcium stress conditions. In the wild-type strain, *SREA* expression was induced upon calcium treatment, and this induction was further enhanced in the *crz1* deletion mutant compared to the wild-type strain (Fig. 3D). Conversely, *CRZ1* expression was significantly lower in the *sreA* deletion mutant than in the wild-type strain under control conditions. However, upon calcium treatment, induction of *CRZ1* was substantially higher in the *sreA* (13-fold) than in the wild-type strain (fivefold). These findings indicate that while SreA and Crz1 operate through distinct pathways, they may exhibit reciprocal transcriptional compensation to mitigate defects in calcium homeostasis.

### SreA affects cytosolic Ca^2+^ dynamics in response to calcium stimulation

Based on the observation that *CRZ1* expression was altered in the *sreA* mutant even under non-stress conditions, we hypothesized that SreA may play a role in regulating early cytosolic Ca^2+^ dynamics. To test this, we monitored changes in cytosolic Ca^2+^ levels in the wild-type, *sreA* mutant, and the complementation strains using Fluo-8 AM following calcium treatment.

**Fig 4:**
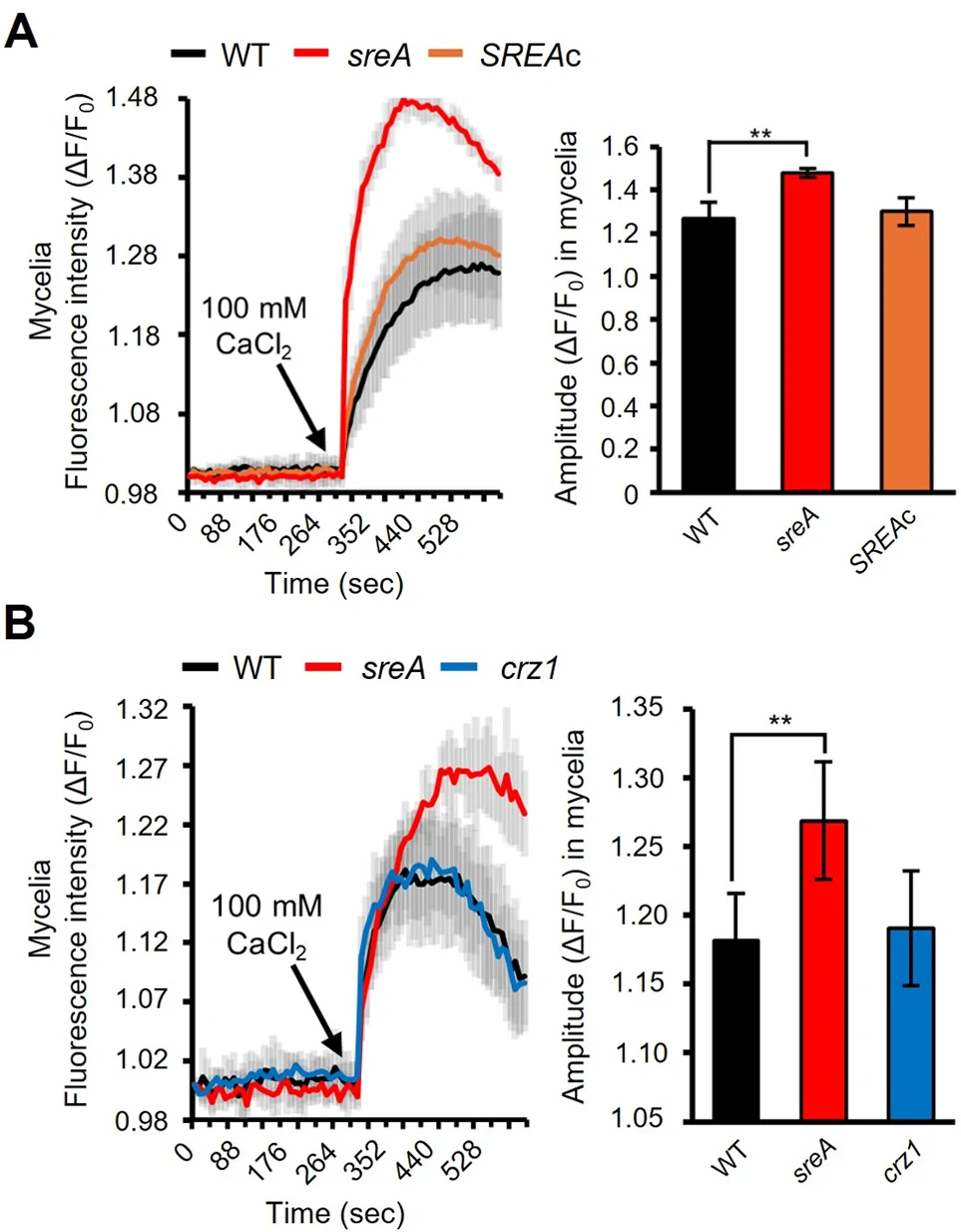
Time-course measurements of cytosolic Ca²^+^ levels in response to calcium treatment. Measurements were performed using fluorescent calcium indication Fluo-8 AM in (**A**) wild-type, *sreA*, and complementation strain (*SREA*c), and (**B**) *crz1* strain. Error bars represent standard deviation. Statistical significance was determined by an unpaired *t*-test (**P < 0.01).

In the wild-type strain, the addition of Ca^2+^ elicited a rapid and transient increase in fluorescence intensity, reflecting a sharp rise in cytosolic Ca^2+^, followed by a gradual return toward basal levels (Fig. 4A). Notably, the amplitude of the cytosolic Ca^2+^ transient ([Ca²^+^]_c_) was significantly elevated in the *sreA* mutant compared to the wild-type strain. This elevated amplitude was fully restored to wild-type levels in the complementation strain. In contrast, the *crz1* mutant exhibited cytosolic Ca^2+^ dynamics comparable to those of the wild type (Fig. 4B), with no significant difference in the peak [Ca²^+^]_c_ amplitude. These results suggest that deletion of *SREA* alters the early cytosolic Ca^2+^ response to calcium stimulation, whereas deletion of *CRZ1* does not affect this response.

## DISCUSSION

Calcium homeostasis is essential for fungal growth and adaptation to environmental stresses and is primarily controlled by the conserved calcineurin–Crz1 pathway. Nevertheless, additional regulatory factors are required to maintain fungal calcium homeostasis. Here, we demonstrate that SreA is also required for calcium stress tolerance in *F. graminearum*.

SreA is an iron-responsive GATA transcription factor that represses genes involved in iron uptake under iron-replete conditions and thereby maintains cellular iron homeostasis in fungi (34–37). Because of this established role, the calcium-sensitive phenotype of the *sreA* mutant raised the possibility that altered iron homeostasis might contribute to the observed defect. This possibility is supported by previous studies suggesting a relationship between calcium and iron homeostasis in fungi. For example, calcium supplementation under iron-deficient conditions suppresses the expression of iron uptake genes in *A. fumigatus* (38). In addition, the transcription factor ZnfA, which contributes to calcium stress tolerance and cooperates with CrzA in the cell wall stress response, directly regulates iron homeostasis genes during caspofungin-induced cell wall stress (21). Consistent with the established role of SreA in iron homeostasis, deletion of *SREA* resulted in excessive intracellular iron accumulation accompanied by increased expression of iron uptake-related genes. However, our analyses indicate that the calcium-sensitive phenotype of the *sreA* mutant cannot be explained by disrupted iron homeostasis. Overexpression of *SIDA*, which promotes siderophore-mediated iron uptake and increases intracellular iron levels, did not alter calcium stress tolerance, indicating that elevated intracellular iron alone does not account for calcium sensitivity. In contrast, the *HAPX* overexpression strain exhibited a mild calcium-sensitive phenotype. Because HapX negatively regulates *SREA* expression, this phenotype is more likely associated with reduced *SREA* expression than with increased intracellular iron accumulation. Collectively, these findings suggest that SreA contributes to calcium stress tolerance that is distinct from its established function in iron homeostasis.

To further define the role of SreA within the established calcium homeostasis network, we examined its genetic and functional relationship with the canonical calcineurin–Crz1 pathway. Multiple lines of evidence indicate that SreA functions independently of the calcineurin and in parallel with Crz1 during the calcium stress response. The enhanced calcium sensitivity of the *sreA crz1* double deletion mutant, together with the lack of reciprocal complementation between SreA and Crz1, demonstrates that these regulators make distinct, non-redundant contributions to calcium stress tolerance. This conclusion is further supported by the constitutive nuclear localization of SreA regardless of calcium treatment or calcineurin inhibition, whereas Crz1 underwent calcineurin-dependent nuclear translocation.

The calcium flux analysis provides further insight into the functional relationship between SreA and Crz1. Deletion of *SREA* resulted in altered cytosolic Ca^2+^ dynamics following calcium stimulation, indicating that SreA contributes to the establishment of appropriate calcium signals during the early phase of the calcium response. Because cytosolic Ca^2+^ signals encode downstream information through their amplitude, duration, and temporal dynamics (39), disruption of these signaling properties is expected to affect calcium-responsive regulatory pathways. Consistent with this interpretation, basal *CRZ1* expression was significantly reduced in the *srea* mutant, whereas *SREA* expression was unaffected by deletion of *CRZ1*. This expression pattern is therefore more consistent with an indirect consequence of disturbed calcium homeostasis than with direct transcriptional regulation between the two transcription factors. Collectively, these findings suggest that SreA contributes to fungal calcium homeostasis by maintaining appropriate cytosolic Ca^2+^ signaling, whereas Crz1 primarily functions downstream as a calcium-responsive transcription factor.

In conclusion, our findings identify SreA as a previously unrecognized regulator of calcium homeostasis in *F. graminearum*. Rather than functioning through the canonical calcineurin–Crz1 pathway, SreA appears to contribute to calcium homeostasis by maintaining appropriate cytosolic Ca^2+^ signaling during the early calcium response. These findings expand our understanding of the mechanisms regulating fungal calcium homeostasis.

## MATERIALS AND METHODS

### Strains and culture conditions

The *F. graminearum* wild-type strain Z-3639 (40) was used as the parental strain for all experiments. All strains, including the wild type and transformants, were maintained on CM agar plates at 25°C and stored as mycelial suspensions in 20% glycerol at −80°C. Culture media were prepared according to the *Fusarium* laboratory manual (41).

Conidiation was induced in either carboxymethyl cellulose medium or on yeast malt agar. To obtain mycelia, conidial suspensions (5 × 10^6^ conidia/mL) were inoculated into liquid CM and incubated for 24 h on a rotary shaker at 200 rpm.

MMM (1.0 g KH_2_PO_4_, 0.5 g MgSO_4_∙7 H_2_O, 0.5 g KCl, 2.0 g NaNO_3_, 30 g sucrose, 0.2 mL trace elements, 10 mL vitamin stock solution, 15 g micro agar per liter) was used as the basal medium for growth and calcium stress assays. All media were prepared using deionized water. Trace element and vitamin stock solutions were filter-sterilized using a 0.22 μm syringe filter and added to the medium after autoclaving. For iron-free conditions (MMM-Fe), Fe(NH₄)₂(SO₄)₂·6H₂O was omitted from the trace element solution. For calcium stress assays, strains were first activated on CM from carnation leaf agar stored at 4°C or from glycerol stocks at –80°C. After 1–2 days of growth on CM, mycelial plugs were transferred to MMM and incubated for an additional 2–3 days. Mycelia subcultured on MMM were subsequently used as the inocula for all calcium stress experiments.

### Nucleic acid manipulation and genetic modification

The TF deletion mutants used in this study, previously generated by Son et al. (32), were further validated by Southern blot analysis to confirm precise gene deletion. Fungal genomic DNA was extracted from lyophilized mycelia as previously described (41). Standard protocols were followed for restriction endonuclease digestion and agarose gel electrophoresis (42). Southern blot hybridization was performed using the North2South Chemiluminescent Hybridization and Detection Kit (Thermo Scientific, Waltham, MA, USA). Oligonucleotide probes were labeled with the North2South Biotin Random Prime DNA Labeling Kit (Thermo Scientific) according to the manufacturer’s instructions.

For the complementation of deletion mutants, GFP-fusion constructs were generated using the yeast gap repair approach (43). The open reading frame and its native promoter regions were amplified using specific primer pairs. The resulting fragments, along with the *Xho*I-digested pDL2 vector, were co-transformed into the yeast strain PJ69-4A for homologous recombination. Transformants were selected based on tryptophan prototrophy. Recombinant plasmids were isolated from yeast, transferred into *Escherichia coli* DH10B, and validated by PCR. To generate overexpression constructs, open reading frames were amplified using the RP27 promoter-specific primer and transformed into yeast as described above. All recombinant plasmids were further confirmed by DNA sequencing.

The fusion PCR product or recombinant vector was introduced into *F. graminearum* protoplasts via PEG-mediated transformation, as previously described (44). Transformants were verified by Southern blot hybridization or qRT-PCR (Fig. S3). All primers used in this study were synthesized by Bioneer (Daejeon, Republic of Korea) and are listed in Table S1. DNA sequencing was performed by Macrogen Inc. (Seoul, Republic of Korea).

### Quantitative reverse transcription-PCR

Total RNA was extracted from fungal mycelia using the Easy-Spin Total RNA Extraction Kit (iNtRON Biotechnology, Seoul, Republic of Korea). First-strand cDNA was synthesized using the SuperScript III First-Strand Synthesis System (Invitrogen, Carlsbad, CA, USA). qRT-PCR was performed using iTaq SYBR Green Master Mix (Bio-Rad, Hercules, CA, USA) and a CFX Real-Time PCR System (Bio-Rad). Endogenous housekeeping genes, ubiquitin C-terminal hydrolase (*CYP1*) and elongation factor 1α (*EF1α*), were used as internal controls for normalization. Primer sets used for qRT-PCR are listed in Table S1. Relative gene expression levels were calculated using the 2^−ΔΔCT^ method (45).

### Strain construction via sexual outcrossing

For outcrossing to construct specific transformants, a heterothallic female strain was fertilized with 1 mL of conidial suspension (1 × 10^6^ conidia/mL) from the designated male strain at 5 days post-inoculation on carrot agar (41). Following fertilization, all cultures were incubated at 25°C for 7–10 days under near-ultraviolet light (365 nm; HKiv Import and Export Co., Ltd., Xiamen, China) to promote ascospore development. Ascospores were then isolated, and the intended genetic modifications were verified either by PCR or phenotyping screening.

### Intracellular [Ca^2+^]_c_ measurement

Measurement of the free Ca^2+^ concentration was performed as previously described with minor modifications (46). Cytosolic Ca^2+^ ([Ca^2+^]_c_) levels were measured in real time using the fluorescent Ca^2 +^ indicator Fluo-8AM (ab142773, Abcam, Cambridge, MA, USA; excitation/emission = 490/520 nm) in a Synergy HTX fluorescence microplate reader (BioTek, Winooski, VT, United States). To minimize fluorescence crosstalk, assays were conducted in clear-bottom, black-walled 96-well plates (#33396, SPL, Pocheon-si, Gyeonggi-do, Republic of Korea). Conidial suspensions (5 × 10^6^ conidia/mL) were inoculated into liquid MMM and cultured at 25°C for 24 h with shaking at 150 rpm. Fungal mycelia were harvested using a spin column (#66900, SPL) and washed three times with 1× PBS. Mycelia were then incubated in a loading buffer containing 4 μM Fluo-8AM, 1% Pluronic F-127 (47), and 2.5 mM probenecid (48) for 2 h at 25°C in the dark. After loading, the mycelia were washed three times with 1× PBS.

Baseline fluorescence was recorded every 8 s for a 5 min period prior to the addition of a specified concentration of CaCl₂. For calcium flux quantification, raw fluorescence intensity values were normalized as previously described (46). Briefly, the average fluorescence intensity before stimulation (*F*₀) was established as the baseline (value = 1). Subsequent fluorescence values (*F*) obtained after CaCl₂ treatment were adjusted relative to this baseline. The results are expressed in terms of the relative fluorescence change (*F*/*F*₀).

### Microscopic observation

Microscopic observation was performed using a Leica DM6 B microscope (Leica Microsystems, Wetzlar, Germany). For GFP observation, an L5 filter set (excitation 480/40 nm; emission 527/30 nm) was used, while an N2.1 filter set (excitation 515–60 nm; emission 590 nm) was employed for red fluorescent protein detection.

To visualize intracellular labile iron levels, each strain was cultured in CM for 24 h and then stained with 5 μM FeRhoNox-1 (Goryo Chemical, Inc., Sapporo, Japan) for 1 h, as previously described (33, 37). Fluorescence signals were captured using the DM6 B microscope with an L5 filter set under consistent exposure times to allow for comparative analysis. All images were processed and analyzed using the Leica Application Suite (LAS) X.

## Data Availability

The data supporting the findings of this study are available on request from the corresponding author.

## SUPPLEMENTAL MATERIAL

Fig. S1 to S3; Table S1 spectrum.01537-26-s0001.pdf Fig. S1: Phenotypic analysis of calcium stress-sensitive strains. Fig. S2: Quantification of iron accumulation in the wild-type, sreA, SIDAoe, and HAPXoe strains. Fig. S3: Validation of genetic modifications in *Fusarium graminearum* strains. Table S1: Primers used in this study.

ASM does not own the copyrights to Supplemental Material that may be linked to, or accessed through, an article. The authors have granted ASM a non-exclusive, world-wide license to publish the Supplemental Material files. Please contact the corresponding author directly for reuse.
