# Supplementary material for: Iron-responsive transcription factor SreA regulates calcium homeostasis independently of calcineurin–Crz1 pathway in Fusarium graminearum
Source: Microbiol Spectr. 2026 Aug 18;14(10):e01537-26. doi: 10.1128/spectrum.01537-26 (PMC13689450; doi:10.1128/spectrum.01537-26)
Supplement: Supplementary file 1 [file spectrum.01537-26-s0001.pdf]

Supplemental Materials

Iron-responsive transcription factor SreA regulates calcium homeostasis independently of calcineurin–Crz1 pathway in *Fusarium graminearum*

Heeji Moon[a#], Jiyeun Park[b#], Soobin Shin[a,c], Sieun Kim[d], Jung-Eun Kim[e], and Hokyoung Son[a,b,f,g*]

[a]*Department of Agricultural Biotechnology, Seoul National University, Seoul, 08826, Republic of Korea*

[b]*Research Institute of Agriculture and Life Sciences, Seoul National University, Seoul, 08826, Republic of Korea*

[c]*The Sainsbury Laboratory, Norwich, NR4 7UH, United Kingdom*

[d]*Agricultural Microbiology Division, National Institute of Agricultural Sciences, Rural Development Administration, Wanju, 55365, Republic of Korea*

[e]*Horticultural and Herbal Crop Environment Division, National Institute of Horticultural and Herbal Science, Rural Development Administration, Wanju, 55365, Republic of Korea*

[f]*Plant Health Center, Seoul National University, Seoul, 08826, Republic of Korea*

[g]*Plant Genomics and Breeding Institute, Seoul National University, Seoul, 08826, Republic of Korea*

Running title: SreA regulates calcium homeostasis in *Fusarium graminearum*

[#] Heeji Moon and Jiyeun Park contributed equally to this work. Author order was determined based on their contribution to the writing of the paper.

[*]Corresponding author: Hokyoung Son, hogongi7@snu.ac.kr

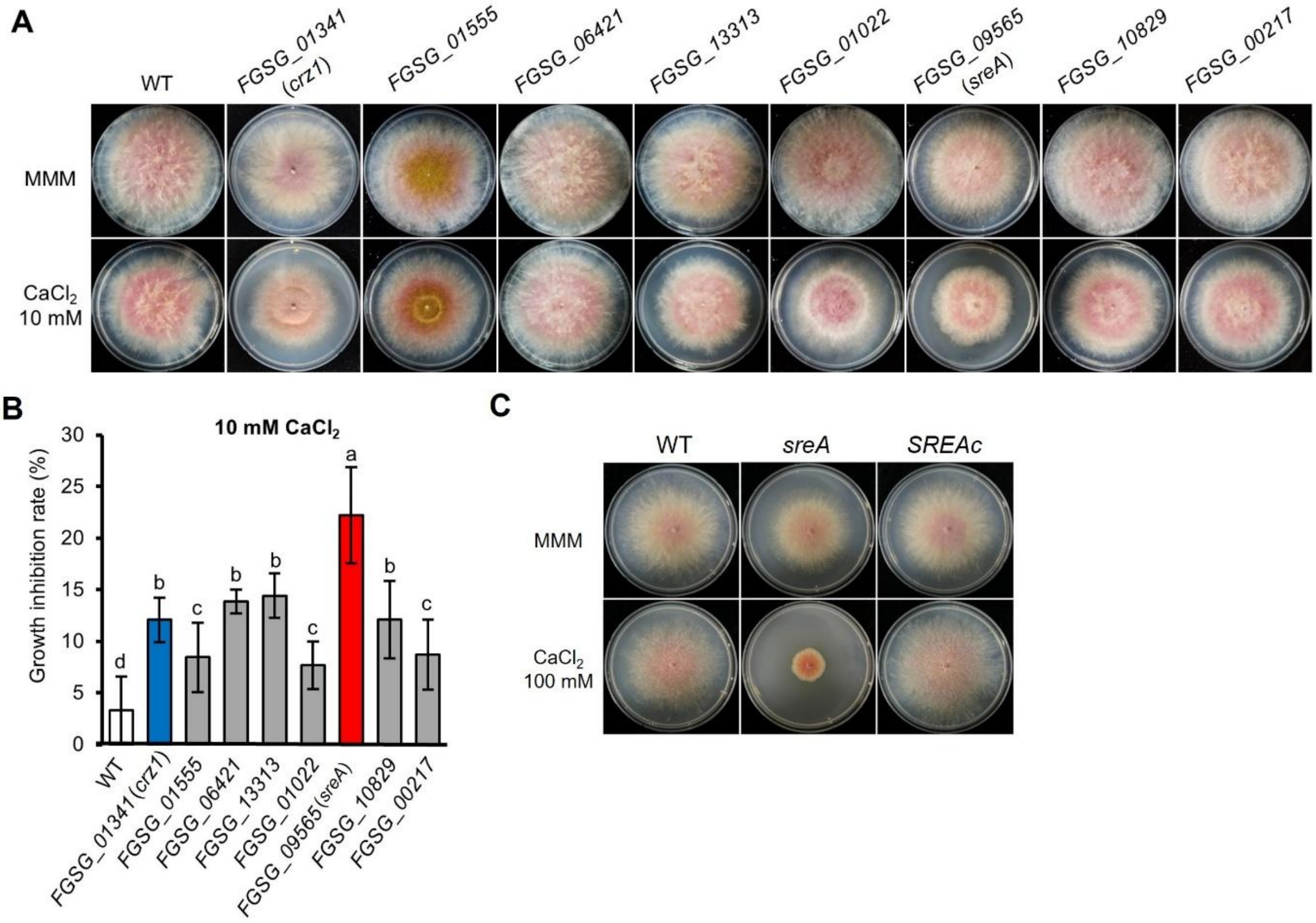

Fig. S1. Phenotypic analysis of calcium stress-sensitive strains. (A) Mycelial growth of eight transcription factor mutants under calcium stress. Each strain was inoculated onto MMM and MMM supplemented with 10 mM $CaCl_2$. Photographs were taken at 5 days post-inoculation. (B) Statistical analysis of mycelial growth inhibition under calcium stress. Different letters indicate significant differences between strains based on one-way analysis of variance (ANOVA), followed by the Bonferroni post-hoc test ($P < 0.01$). Data represent the mean ± standard deviation from at least three independent replicates. (C) Mycelial growth of the wild-type, *sreA*, and complemented strains on MMM and MMM supplemented with 100 mM $CaCl_2$. Photographs were taken at 5 days post-inoculation.

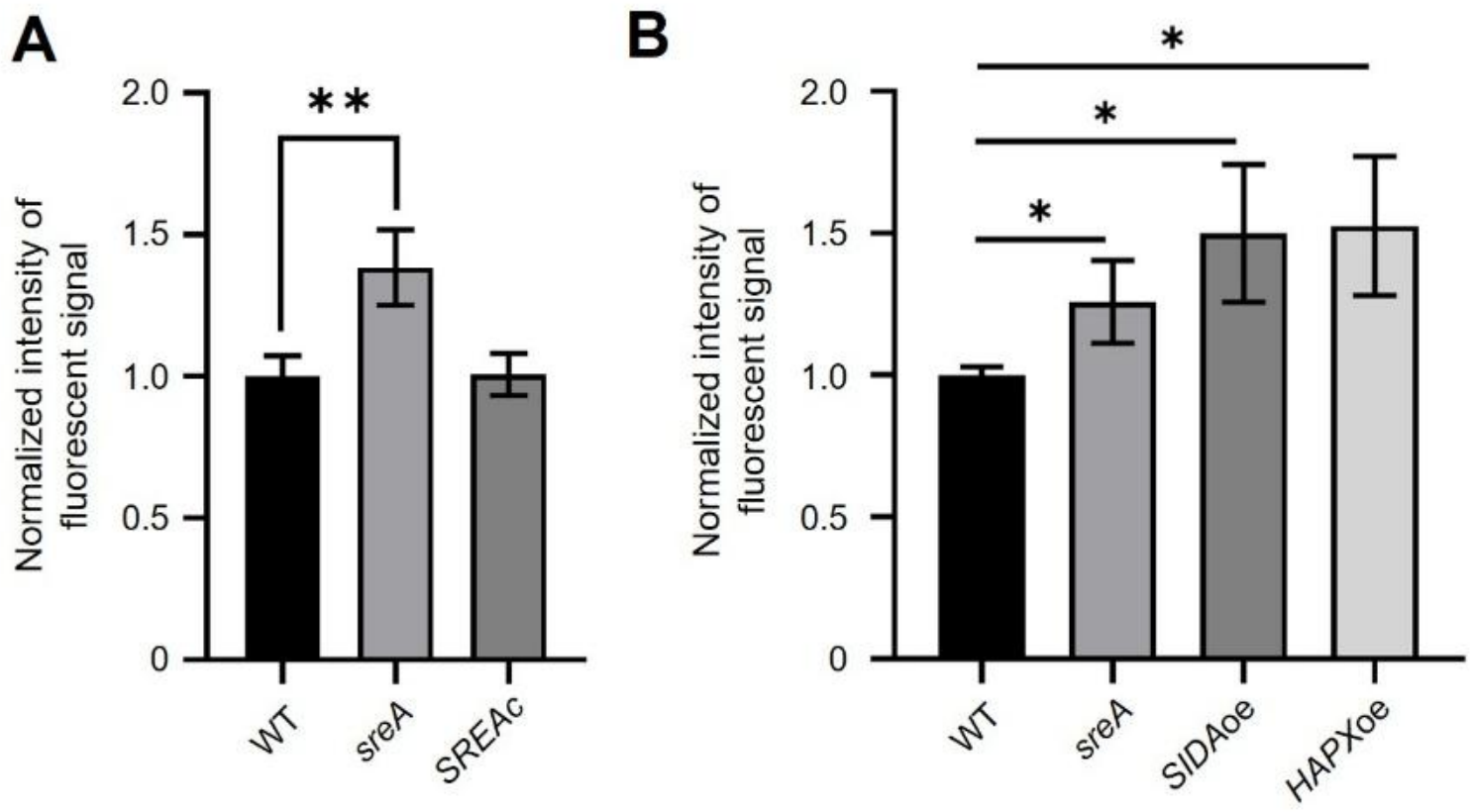

Fig. S2. Quantification of iron accumulation in the wild-type, *sreA*, *SIDA*oe, and *HAPX*oe strains. Iron accumulation was detected using FeRhoNox-1, and fluorescence intensity was quantified using ImageJ. Statistical significance was determined using Student's *t*-test (* $p < 0.05$, ** $p < 0.005$).

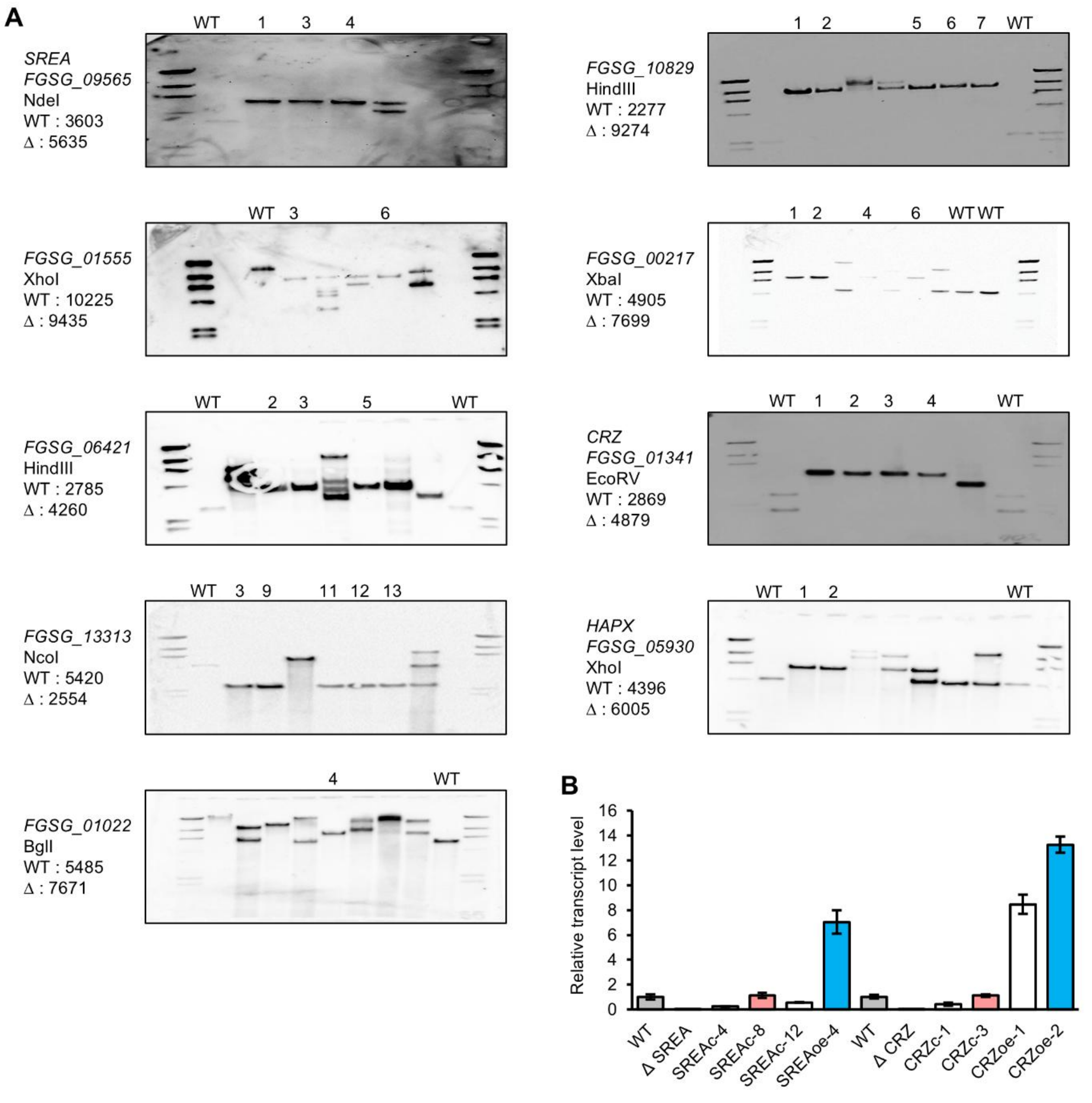

Fig. S3. Validation of genetic modifications in *Fusarium graminearum* strains. (A) Southern blot analysis for the confirmation of transcription factor (TF) and *HAPX* deletion mutants. The expected DNA fragment sizes for the wild type and each mutant are indicated to the left of each of the nine panels. (B) Verification of complementation and overexpression strains using qRT-PCR. Relative transcript levels of the target genes were measured to confirm the successful restoration of gene expression in complementation strains and increased expression in overexpression strains. Data represent the mean ± standard deviation from three independent biological replicates.

Table S1. Primers used in this study

Primer name	Sequence (5'→3')	Description
FgSidA-RT-F	TGCTGTTATTGGTGCCGGTC	For qRT-PCR
FgSidA-RT-R	AGCCTCGGTAAGCAGTGTCT	
FgMirB-RT-F	CACAAGGCGAAGAAGAATAT	
FgMirB-RT-R	AGGACGAAACCAACTACAAT	
FgSidC-RT-F	CAACAGCGGATGCGATTCTC	
FgSidC-RT-R	TCTCAAACACGCTCACACTC	
FgSidD-RT-F	TCACCATCATTTTCATCTCC	
FgSidD-RT-R	ACTTTCTCTATGTCTTGCTC	
SREA-RT-F	GATGGCAGCCAACGATGATAGA	
SREA-RT-R	GGCTGACATTGAGGGGTTGAGA	
CRZ-RT-F	ACGCTCTTGCTCGCCACTCTA	
CRZ-RT-R	ACTCTTCTTCGTCGGCGTTATCA	
CYP1-RT-F	TCAAGCTCAAGCACACCAAGAAGG	
CYP1-RT-R	GGTCCGCCGCTCCAGTCT	
EF1A-RT-F	GGCTTTCACCGACTACCCTCCTCT	
EF1A-RT-R	ACTTCTCGACGGCCTTGATGACAC	
SREAoe-F	GGAGGCGGAGGCGGAGGCTTAATTAACATGAGCCAAGAAGCCCAAACAG	Amplification of the *SREA* gene fragment for overexpression
SREAoe-R	CCTGAATGTTGAGTGGAATGATCTCGAGTTAATCGTGGCCAAGTTCTCGTAG	
SREAcom-F	CCCGGTGAACAGCTCCTCGCCCTTGCTCACATCGTGGCCAAGTTCTCGTAGC	Amplification of the *SREA* gene fragment for complementation
SREAcom-R	TATAGGGCGAATTGGGTACTCAAATTGGTTTTACACGGTAGACAGACAGGCACTT	
CRZcom-F	TATAGGGCGAATTGGGTACTCAAATTGGTTTTGCACGCAAATCATCATCAGTTA	Amplification of the *CRZ1* gene fragment for complementation & overexpression
CRZoe-F	TTTCGTAGGAACCCAATCTTCAAACAGCCACATGTGAGAGCAGAGAAA	
CRZcom&oe-R	CCCGGTGAACAGCTCCTCGCCCTTGCTCACCCGGCCACCAAAGTCACTG	
HapXoe-F	TTTCGTAGGAACCCAATCTTCAAACATGTCGGCATCTTCTCCTCAAC	Amplification of the *HAPX* gene fragment for overexpression
HapXoe-R	CCCGGTGAACAGCTCCTCGCCCTTGCTCACAATTCCTCTCCCAAACCGAACG	
SidAoe-F	TTTCGTAGGAACCCAATCTTCAAAATGAGTCCCCACAGCGAGATTT	Amplification of the *SIDA* gene fragment for overexpression
SidAoe-R	CCCGGTGAACAGCTCCTCGCCCTTGCTCACCTGCTTGAAAATAGACTGAACTATCTCG	
